# Supplementary material for: Current trends in the application of causal inference methods to pooled longitudinal observational infectious disease studies—A protocol for a methodological systematic review
Source: PLoS One. 2021 Apr 29;16(4):e0250778. doi: 10.1371/journal.pone.0250778 (PMC8084147; doi:10.1371/journal.pone.0250778)
Supplement: S1 Table — (PDF) [file pone.0250778.s002.pdf]

Attachment 1. Search strategy

1. EBSCO (PsycINFO, Academic Search Complete, Business Source Premier, CINAHL, EconLit with Full Text)
2. EMBASE
3. PubMed
4. Web of Science

1. EBSCO-- Academic Search Ultimate, Business Source Premier, CINAHL, EconLit with Full Text, APA PyscINFO

#	Searches
1	AB ("individual patient data" OR "individual participant data" OR "IPD" OR “individual-patient-data” OR “individual-participant-data” OR “individual-participant” OR “individual-patient” OR “participant data” OR “patient data” OR “individual-level”)
2	TI (("individual patient data" OR "individual participant data" OR "IPD" OR “individual-patient-data” OR “individual-participant-data” OR “individual-participant” OR “individual-patient” OR “participant data” OR “patient data” OR “individual-level”)
3	AB (((“cohort”) OR (“longitudinal”)) N4 ((pool*) OR (aggregat*) OR (harmoniz*)))
4	TI (((“cohort”) OR (“longitudinal”)) N4 ((pool*) OR (aggregat*) OR (harmoniz*)))
5	S1 OR S2 OR S3 OR S4
6	SU (infectious disease OR myelitis OR anaplasmosis OR babesiosis OR botulism OR Brucellosis OR Campylobacter Infections ORchancroid OR chikungunya fever OR chikungunya virus OR chlamydia OR Ciguatera Poisoning OR clostridium infections OR coronavirus infections OR Creutzfeldt-Jakob Syndrome OR cryptosporidiosis OR dengue virus OR dengue OR diphtheria OR escherichia coli OR encephalomyelitis disseminata OR ebola virus OR ehrlichiosis OR Encephalitis Viruses OR Enterovirus Infections OR giardiasis OR glanders OR Gonorrhea OR Herpes Simplex OR herpes labialis OR herpes genitalis OR histoplasma OR hiv OR papillomavirus infections OR Influenza, Human OR influenza virus OR legionellosis OR leprosy OR leptospira OR listeriosis OR lyme disease OR malaria OR Measles OR histoplasma OR hiv OR papillomavirus infections OR Influenza, Human OR influenza virus OR legionellosis OR leprosy OR leptospira OR listeriosis OR lyme disease OR malaria OR measles OR melioidosis OR (mers or middle east respiratory syndrome) OR mumps OR norovirus OR pediculosis OR whooping cough OR plague OR streptococcal infection OR poliomyelitis OR lyme disease OR psittacosis OR Phthirus OR smallpox OR rabies OR rickettsia OR rubella OR salmonella OR scabies OR sars virus OR dysentery OR (methicillin-resistant staphylococcus aureus or mrsa) OR syphilis OR trichomonas OR (tuberculosis OR tb) OR tularemia OR typhoid fever OR typhus OR chickenpox OR cholera OR vibrio OR hemorrhagic fever OR flavivirus OR yellow fever OR yersinia OR zika virus OR arboviruses)
7	TI (infectious disease OR myelitis OR anaplasmosis OR babesiosis OR botulism OR Brucellosis OR Campylobacter Infections ORchancroid OR chikungunya fever OR chikungunya virus OR chlamydia OR Ciguatera Poisoning OR clostridium infections OR coronavirus infections OR Creutzfeldt-Jakob Syndrome OR cryptosporidiosis OR dengue virus OR dengue OR diphtheria OR escherichia coli OR encephalomyelitis disseminata OR ebola virus OR ehrlichiosis OR Encephalitis Viruses OR Enterovirus Infections OR giardiasis OR glanders OR Gonorrhea OR Herpes Simplex OR herpes labialis OR herpes genitalis OR histoplasma OR hiv OR papillomavirus infections OR Influenza, Human OR influenza virus OR legionellosis OR leprosy OR leptospira OR listeriosis OR lyme disease OR malaria OR Measles OR histoplasma OR hiv OR papillomavirus infections OR Influenza, Human OR influenza virus OR legionellosis OR leprosy OR leptospira OR listeriosis OR lyme disease OR malaria OR measles OR melioidosis OR (mers or middle east respiratory syndrome) OR mumps OR norovirus OR pediculosis OR whooping cough OR plague OR streptococcal infection OR poliomyelitis OR lyme disease OR psittacosis OR Phthirus OR smallpox OR rabies OR rickettsia OR rubella OR salmonella OR scabies OR sars virus OR dysentery OR (methicillin-resistant staphylococcus aureus or mrsa) OR syphilis OR trichomonas OR (tuberculosis OR tb) OR tularemia OR typhoid fever OR typhus OR chickenpox OR cholera OR vibrio OR hemorrhagic fever OR flavivirus OR yellow fever OR yersinia OR zika virus OR arboviruses)
8	S6 OR S7
9	S5 AND S8

10	TI ("single center" OR "single centre" OR "single-centre" OR "single-center" OR “multi center” OR “multi centre” OR “multi-centre” OR “multi-center” OR “multicenter” OR “multicentre” OR “multi-site” OR “cross-sectional” OR “cross sectional” OR “predict*” OR “prognos*” OR “protocol” OR “erratum” or “correction”)
11	S9 NOT S10
12	SU (randomized controlled trials OR rtc OR randomised control trial OR randomized control trial OR randomized clinical trial OR randomised clinical trial OR randomized controlled study OR animal experimentation or animal testing or animal research)
13	S11 NOT S12
14	SO editorial or opinion or commentary
15	S13 NOT S14
16	Limit publication years: 2009, 2014, 2019
17	Limit 16 to English

2. Embase

#	Searches
1	individual NEAR/4 level NEAR/4 data
2	individual NEAR/4 patient NEAR/4 data
3	'individual patient data'/exp
4	individual NEAR/4 participant NEAR/4 data
5	#1 OR #2 OR #3 OR #4
6	cohort OR longitudinal
7	pool* OR harmoniz* OR aggregat*
8	#6 OR #7
9	#5 OR #8
10	'myelitis'/exp OR 'anaplasmosis'/exp OR 'piroplasmosis'/exp OR 'botulism'/exp OR 'brucellosis'/exp OR 'campylobacter'/exp OR 'ulcus molle'/exp OR 'chikungunya'/exp OR 'chikungunya virus'/exp OR 'chlamydia'/exp OR 'ciguatera'/exp OR 'clostridium infection'/exp OR 'coronavirus infection'/exp OR 'creutzfeldt jakob disease'/exp OR 'cryptosporidiosis'/exp OR 'dengue virus'/exp OR 'dengue'/exp OR 'diphtheria'/exp OR 'escherichia coli'/exp OR 'eastern equine encephalitis'/exp OR 'ebolavirus'/exp OR 'ehrlichiosis'/exp OR 'encephalitis virus'/exp OR 'enterovirus infection'/exp OR 'giardiasis'/exp OR 'glanders'/exp OR 'gonorrhea'/exp OR 'herpes simplex virus'/exp OR 'herpes labialis'/exp OR 'genital herpes'/exp OR 'histoplasmosis'/exp OR 'human immunodeficiency virus'/exp OR 'papillomavirus infection'/exp OR 'influenza virus'/exp OR 'legionnaire disease'/exp OR 'leprosy'/exp OR 'listeriosis'/exp OR 'lyme disease'/exp OR 'malaria'/exp OR 'measles'/exp OR 'melioidosis'/exp OR 'middle east respiratory syndrome coronavirus'/exp OR 'mumps'/exp OR 'norovirus'/exp OR 'pediculus'/exp OR 'pertussis'/exp OR 'plague'/exp OR 'streptococcus infection'/exp OR 'poliomyelitis'/exp OR 'tick borne encephalitis'/exp OR 'ornithosis'/exp OR 'smallpox'/exp OR 'rabies'/exp OR 'rickettsiosis'/exp OR 'rubella'/exp OR 'salmonella'/exp OR 'scabies'/exp OR 'sars-related coronavirus'/exp OR 'methicillin resistant

	staphylococcus aureus'/exp OR 'syphilis'/exp OR 'trichomoniasis'/exp OR 'tuberculosis'/exp OR 'tularemia'/exp OR 'typhoid fever'/exp OR 'typhus'/exp OR 'varicella zoster virus'/exp OR 'cholera'/exp OR 'vibriosis'/exp OR 'hemorrhagic fever'/exp OR 'flavivirus infection'/exp OR 'yellow fever virus'/exp OR 'yersinia infection'/exp OR 'zika virus'/exp OR 'arbovirus'/exp
11	#9 AND #10
12	single AND center:ti OR (single AND centre:ti) OR 'single centre':ti OR 'single center':ti OR (multi AND center:ti) OR 'multi center':ti OR multicenter:ti OR multicentre:ti OR 'multi site':ti OR 'cross sectional':ti OR crosssectional:ti OR predict*:ti OR prognos*:ti OR (randomized AND control*:ti) OR (randomised AND control*:ti) OR (randomized AND clinical:ti) OR (randomised AND clinical:ti) OR (randomized AND trial:ti) OR (randomised AND trial:ti) OR rct:ti OR (clinical AND trial:ti) OR protocol:ti OR erratum:ti OR correction:ti
13	#11 NOT #12
14	'randomized controlled trial' OR 'clinical study'
15	'animal' NOT 'human'
16	#14 OR #15
17	#13 NOT #16
18	#17 AND ('article'/it OR 'article in press'/it) AND (2009:py OR 2014:py OR 2019:py) AND [english]/lim
1	individual NEAR/4 level NEAR/4 data
2	individual NEAR/4 patient NEAR/4 data
3	'individual patient data'/exp

3. Pubmed

1	"Individual patient data"[Title/Abstract] OR "individual participant data"[Title/Abstract] OR "ipd"[Title/Abstract] OR "individual-patient-data"[Title/Abstract] OR "individual-participant-data"[Title/Abstract] OR “participant data”[Title/Abstract] OR “patient data”[Title/Abstract] OR “individual-participant”[Title/Abstract] OR “individual-patient”[Title/Abstract] OR “individual-level”[Title/Abstract]
2	("cohort"[Title/Abstract] OR "longitudinal"[Title/Abstract]) AND ("pool*"[Title/Abstract] OR “aggregat*"[Title/Abstract] OR "harmoniz*”[Title/Abstract])
3	#1 OR #2

4	((("Communicable Diseases"[Mesh]) OR "Myelitis"[Mesh]) OR "Anaplasmosis"[Mesh]) OR "Babesiosis"[Mesh]) OR "Botulism"[Mesh]) OR "Brucellosis"[Mesh]) OR "Campylobacter Infections"[Mesh]) OR "Chancroid"[Mesh]) OR "Chikungunya Fever"[Mesh]) OR "Chikungunya virus"[Mesh]) OR "Chlamydia"[Mesh]) OR "Ciguatera Poisoning"[Mesh]) OR "Clostridium Infections"[Mesh]) OR "Coronavirus Infections"[Mesh]) OR "Creutzfeldt-Jakob Syndrome"[Mesh]) OR "Cryptosporidiosis"[Mesh]) OR "Dengue Virus"[Mesh]) OR "Dengue"[Mesh]) OR "Diphtheria"[Mesh]) OR "Escherichia coli Proteins"[Mesh]) OR "Encephalomyelitis, Eastern Equine"[Mesh]) OR "Ebolavirus"[Mesh]) OR "Ehrlichiosis"[Mesh]) OR "Encephalitis Viruses"[Mesh]) OR "Enterovirus Infections"[Mesh]) OR "Giardiasis"[Mesh]) OR "Glanders"[Mesh]) OR "Gonorrhea"[Mesh]) OR "Herpes Simplex"[Mesh]) OR "Herpes Labialis"[Mesh]) OR "Herpes Genitalis"[Mesh]) OR "Histoplasma"[Mesh]) OR "HIV"[Mesh]) OR "Papillomavirus Infections"[Mesh]) OR "Influenza, Human"[Mesh]) OR "Influenzavirus A"[Mesh]) OR "Influenza B virus"[Mesh]) OR "Legionellosis"[Mesh]) OR "Leprosy"[Mesh]) OR "Leptospira"[Mesh]) OR "Listeriosis"[Mesh]) OR "Lyme Disease"[Mesh]) OR "Malaria"[Mesh]) OR "Measles virus"[Mesh]) OR "Melioidosis"[Mesh]) OR "Middle East Respiratory Syndrome Coronavirus"[Mesh]) OR "Mumps"[Mesh]) OR "Norovirus"[Mesh]) OR "Pediculus"[Mesh]) OR "Whooping Cough"[Mesh]) OR "Plague"[Mesh]) OR "Streptococcal Infections"[Mesh]) OR "Poliomyelitis"[Mesh]) OR "Encephalitis, Tick-Borne"[Mesh]) OR "Psittacosis"[Mesh]) OR "Phthirus"[Mesh]) OR "Smallpox"[Mesh]) OR "Rabies"[Mesh]) OR "Rickettsia Infections"[Mesh]) OR "Rubella"[Mesh]) OR "Salmonella"[Mesh]) OR "Scabies"[Mesh]) OR "SARS Virus"[Mesh]) OR "Dysentery"[Mesh]) OR "Methicillin-Resistant Staphylococcus aureus"[Mesh]) OR "Syphilis"[Mesh]) OR "Trichomonas Infections"[Mesh]) OR "Tuberculosis"[Mesh]) OR "Tularemia"[Mesh]) OR "Typhoid Fever"[Mesh]) OR "Typhus, Epidemic Louse-Borne"[Mesh]) OR "Typhus, Endemic Flea-Borne"[Mesh]) OR "Chickenpox"[Mesh]) OR "Cholera"[Mesh]) OR "Vibrio Infections"[Mesh]) OR "Hemorrhagic Fevers, Viral"[Mesh]) OR "Flavivirus Infections"[Mesh]) OR "Yellow fever virus"[Mesh]) OR "Yersinia Infections"[Mesh]) OR "Zika Virus Infection"[Mesh]) OR "Arbovirus Infections"[Mesh])
5	((("Communicable Diseases"[Title]) OR "Myelitis"[Title]) OR "Anaplasmosis"[Title]) OR "Babesiosis"[Title]) OR "Botulism"[Title]) OR "Brucellosis"[Title]) OR "Campylobacter Infections"[Title]) OR "Chancroid"[Title]) OR "Chikungunya Fever"[Title]) OR "Chikungunya virus"[Title]) OR "Chlamydia"[Title]) OR "Ciguatera Poisoning"[Title]) OR "Clostridium Infections"[Title]) OR "Coronavirus Infections"[Title]) OR "Creutzfeldt-Jakob Syndrome"[Title]) OR "Cryptosporidiosis"[Title]) OR "Dengue Virus"[Title]) OR "Dengue"[Title]) OR "Diphtheria"[Title]) OR "Escherichia coli Proteins"[Title]) OR "Encephalomyelitis, Eastern Equine"[Title]) OR "Ebolavirus"[Title]) OR "Ehrlichiosis"[Title]) OR "Encephalitis Viruses"[Title]) OR "Enterovirus Infections"[Title]) OR "Giardiasis"[Title]) OR "Glanders"[Title]) OR "Gonorrhea"[Title]) OR "Herpes Simplex"[Title]) OR "Herpes Labialis"[Title]) OR "Herpes Genitalis"[Title]) OR "Histoplasma"[Title]) OR "HIV"[Title]) OR "Papillomavirus Infections"[Title]) OR "Influenza, Human"[Title]) OR "Influenzavirus A"[Title]) OR "Influenza B virus"[Title]) OR "Legionellosis"[Title]) OR "Leprosy"[Title]) OR "Leptospira"[Title]) OR "Listeriosis"[Title]) OR "Lyme Disease"[Title]) OR "Malaria"[Title]) OR "Measles virus"[Title]) OR "Melioidosis"[Title]) OR "Middle East Respiratory Syndrome Coronavirus"[Title]) OR "Mumps"[Title]) OR "Norovirus"[Title]) OR "Pediculus"[Title]) OR "Whooping Cough"[Title]) OR "Plague"[Title]) OR "Streptococcal Infections"[Title]) OR "Poliomyelitis"[Title]) OR "Encephalitis, Tick-Borne"[Title]) OR "Psittacosis"[Title]) OR "Phthirus"[Title]) OR "Smallpox"[Title]) OR "Rabies"[Title]) OR "Rickettsia Infections"[Title]) OR "Rubella"[Title]) OR "Salmonella"[Title]) OR "Scabies"[Title]) OR "SARS Virus"[Title]) OR "Dysentery"[Title]) OR "Methicillin-Resistant Staphylococcus aureus"[Title]) OR "Syphilis"[Title]) OR "Trichomonas Infections"[Title]) OR "Tuberculosis"[Title]) OR "Tularemia"[Title]) OR "Typhoid Fever"[Title]) OR "Typhus, Epidemic Louse-Borne"[Title]) OR "Typhus, Endemic Flea-Borne"[Title]) OR "Chickenpox"[Title]) OR "Cholera"[Title]) OR "Vibrio Infections"[Title]) OR "Hemorrhagic Fevers, Viral"[Title]) OR "Flavivirus Infections"[Title]) OR "Yellow fever virus"[Title]) OR "Yersinia Infections"[Title]) OR "Zika Virus Infection"[Title]) OR "Arbovirus Infections"[Title])
6	#4 OR #5
7	#3 AND #6
8	"single-center"[Title] OR "single-center"[Title] OR "single-centre"[Title] OR "single-centre"[Title] OR "multi-center"[Title] OR "multi-center"[Title] OR "multi-centre"[Title] OR "multi-centre"[Title] OR "multicenter"[Title] OR "multicentre"[Title] OR "multi-site"[Title] OR "multisite"[Title] OR "multiple sites"[Title] OR "cross-sectional"[Title] OR "cross-sectional"[Title] OR "transversal"[Title] OR "prognos*"[Title] OR "predict*"[Title] OR "randomized control*"[Title] OR "randomised control*"[Title] OR "randomised clinical"[Title]

	OR "randomized clinical"[Title] OR "randomized trial*"[Title] OR "randomised trial*"[Title] OR "rct"[Title] OR "protocol"[Title] OR "erratum"[Title] OR "correction"[Title] OR "author correction"[Title]
9	#7 NOT #8
10	"randomized controlled trials as topic"[MeSH Terms] OR "animal experimentation"[MeSH Terms] OR "animal testing alternatives"[MeSH Terms] OR "animal experimentation"[MeSH Terms]
11	#9 NOT #10
12	"address"[Publication Type] OR "autobiography"[Publication Type] OR "bibliography"[Publication Type] OR "biography"[Publication Type] OR "book illustrations"[Publication Type] OR "webcast"[Publication Type] OR "case reports"[Publication Type] OR "clinical trial, veterinary"[Publication Type] OR "collected work"[Publication Type] OR "collected works"[Publication Type] OR "comment"[Publication Type] OR "consensus development conference"[Publication Type] OR "dataset"[Publication Type] OR "dictionary"[Publication Type] OR "directory"[Publication Type] OR "duplicate publication"[Publication Type] OR "editorial"[Publication Type] OR "electronic supplementary materials"[Publication Type] OR "ephemera"[Publication Type] OR "equivalence trial"[Publication Type] OR "evaluation studies"[Publication Type] OR "evaluation study"[Publication Type] OR "expression of concern"[Publication Type] OR "festschrift"[Publication Type] OR "interactive tutorial"[Publication Type] OR "interview"[Publication Type] OR "lecture"[Publication Type] OR "legal case"[Publication Type] OR "legislation"[Publication Type] OR "letter"[Publication Type] OR "news"[Publication Type] OR "newspaper article"[Publication Type] OR "observational study, veterinary"[Publication Type] OR "patient education handout"[Publication Type] OR "periodical index"[Publication Type] OR "personal narrative"[Publication Type] OR "pictorial work"[Publication Type] OR "portrait"[Publication Type] OR "published erratum"[Publication Type] OR "randomized controlled trial"[Publication Type] OR "randomized controlled trial, veterinary"[Publication Type] OR "retracted publication"[Publication Type] OR "retraction of publication"[Publication Type] OR "video-audio media"[Publication Type] OR "validation study"[Publication Type]
13	#11 NOT #12
14	Limit publication years: 2009, 2014, 2019
15	Limit 14 to English

4. Web of Science- Core

#	**Searches**
1	TS=("individual patient data" OR "individual participant data" OR "IPD" OR “individual-patient-data”:ti,ab OR “individual-participant-data” OR “individual-participant” OR “individual-patient” OR “participant data” OR “patient data” OR “individual-level”)
2	TS =((“cohort” OR “longitudinal”) NEAR/4 (pool* OR aggregat* OR harmoniz*))
3	#1 OR #2
4	TS= (“infectious disease*” OR "Myelitis" OR "Anaplasmosis" OR "Babesiosis" OR "Botulism" OR "Brucellosis" OR "Campylobacter Infections" OR "Chancroid" OR "Chikungunya Fever" OR "Chikungunya virus" OR "Chlamydia" OR "Ciguatera Poisoning" OR "Clostridium Infections" OR "Coronavirus Infections" OR "Creutzfeldt-Jakob Syndrome" OR "Cryptosporidiosis" OR "Dengue Virus" OR "Dengue" OR "Diphtheria" OR "Escherichia coli Proteins" OR "Encephalomyelitis, Eastern Equine" OR "Ebolavirus" OR "Ehrlichiosis" OR "Encephalitis Viruses" OR "Enterovirus Infections" OR "Giardiasis" OR "Glanders" OR "Gonorrhea" OR "Herpes Simplex" OR "Herpes Labialis" OR "Herpes Genitalis" OR "Histoplasma" OR "HIV" OR "Papillomavirus Infections" OR "Influenza, Human" OR “Influenza” OR “Flu” "Influenza virus A" OR "Influenza B virus" OR "Legionellosis" OR "Leprosy" OR "Leptospira" OR "Listeriosis" OR "Lyme Disease" OR "Malaria" OR "Measles virus" OR "Melioidosis" OR "Middle East Respiratory Syndrome Coronavirus" OR "Mumps" OR "Norovirus" OR "Pediculus" OR "Whooping Cough" OR "Plague" OR "Streptococcal Infections" OR "Poliomyelitis" OR "Encephalitis, Tick-Borne" OR "Psittacosis" OR "Phthirus" OR "Smallpox" OR "Rabies" OR "Rickettsia Infections" OR "Rubella" OR "Salmonella" OR "Scabies" OR "SARS Virus" OR "Dysentery" OR "Methicillin-Resistant Staphylococcus aureus" OR "Syphilis" OR "Trichomonas Infections" OR "Tuberculosis" OR "Tularemia" OR "Typhoid Fever" OR

	"Typhus, Epidemic Louse-Borne" OR "Typhus, Endemic Flea-Borne" OR "Chickenpox" OR "Cholera" OR "Vibrio Infections" OR "Hemorrhagic Fevers, Viral" OR "Flavivirus Infections" OR "Yellow fever virus" OR "Yersinia Infections" OR "Zika Virus Infection" OR "Arbovirus Infections")
5	#3 AND #4
6	TI=("single center" OR "single centre" OR "single-centre" OR "single-center" OR “multi center” OR “multi centre” OR “multi-centre” OR “multi-center” OR “multicenter” OR “multicentre” OR “multi-site” OR “cross-sectional” OR “cross sectional” OR “predict*” OR “prognos*” OR “protocol” OR “erratum” or “correction”)
7	#5 NOT #6
8	TS=("RCT*" OR "Randomi?ed Controlled Trial*" OR "animal model" OR animal*)
9	#7 NOT #8
10	In document types, exclude meeting abstracts, proceedings paper, letter, correction, book chapter, reprint, book review, news item, note, and retracted publication, editorial material.
11	Limit publication years: 2009, 2014, 2019
12	Limit 11 to English
