# Supplementary material for: Current trends in the application of causal inference methods to pooled longitudinal observational infectious disease studies—A protocol for a methodological systematic review
Source: PLoS One. 2021 Apr 29;16(4):e0250778. doi: 10.1371/journal.pone.0250778 (PMC8084147; doi:10.1371/journal.pone.0250778)
Supplement: S2 Table — (PDF) [file pone.0250778.s003.pdf]

Current Trends in the Application of Causal Inference Methods to Pooled Longitudinal Observational Infectious Disease Studies - Data Extraction Form

**Title of the paper/article/report**
**Study ID** *(surname of first author and year first full report of study was published e.g. Smith 2001)*
**Report IDs of other reports of this study** *(e.g. duplicate publications, follow-up studies)*
Notes:

1. General Information *(Example entries)*

**1. Date form completed (dd/mm/yyyy)**	15/04/2020
**2. Name/ID of person extracting data**	Heather/Saba/Ellie/Lauren
**3. Title of article (title of paper/abstract/report that data are extracted from)**	“Smoking and bmi in 21 twin birth cohorts”
**4. First author**	Donegan, L. M.
**5. Contact Details**	lisa.donegan@fake-uni.edu
**6. Publication type (e.g. full report, abstract, letter)**	Full report
**7. Possible conflicts of interest (for study authors)**	None claimed
**Notes:**	

2. Eligibility to be included in this systematic review (not inclusion/exclusion criteria for the study participants)

Study Characteristics	Copy and paste related descriptions as stated in report/paper	Location in text (pg and paragraph number/fig/table)
**8. Is the parent study composed of 2+ separate observational and longitudinal studies?** *If yes, include.* ***i****f only single-site or multi-site, single-cohort, exclude; if includes RCTs, go to next question.*		
**9. Are any of the included studies RCTs?** *If yes, is the parent study using non-randomized variables from at least one RCT or observational cohort in the analysis? If yes, include. If not, exclude.*		
**10. Does the paper deal with infectious disease?** *If yes, include. If not, exclude.*		
**11. Are study subjects human?** *If animals, or human tissue samples or similar, exclude.*		
**12. Is it focused on health outcomes?** *If not, exclude.No wages, salaries, etc.*		
**13. Does the paper include case studies?** *If yes, exclude.*		
**14. Is it a methods paper with the primary goal of describing, developing, or summarizing a statistical analytical method, with no original analysis of observational, longitudinal data?** *If yes, exclude. If with an applied real-life example, include.*		

**15. Is the paper a protocol, review, commentary correction, editorial, erratum, or similar?** *If yes, exclude*		
**16. Is the paper in English?** *If not, exclude*		
**17. Is the data pooled on an individual level?** *If not, exclude.*		
**18. Does the study attempt to establish a causal relationship?** *E.g. by controlling for confounding variables or using an IV approach. If the goal of the study is descriptive, prediction, or prognostication, exclude. If unsure, flag as unsure.*		
**19. Is an effect size estimated?**		
**20. Is the effect size directly related to the causal question?** *If the effect size does not correspond to the stated causal research question, exclude*		
**21. Is the study published in either 2009, 2014, or 2019?** *If not, exclude.*		
**22. Decision to include**		
**23. Notes**		

DO NOT PROCEED IF STUDY IS EXCLUDED FROM REVIEW

3. Study characteristics

	**Copy and paste related descriptions as stated in report/paper**	**Location in text (pg & paragraph/fig/table)**
**24. List number & types of <u>individual</u> studies or cohorts/clusters included in the pooled analysis** *E.g. 3 cohort studies, 2*		

case-control studies		
**25. Study populations of each individual study or cohort included in the pooled analysis**		
**26. Number of participants in each individual study or cohort included in the pooled analysis**		
**27. Recruitment period of each individual study or cohort included in the pooled analysis**		
**28. Location of data collection of each individual study or cohort included in the pooled analysis**		
**Parent study**		
**29. Discipline of parent study?** (e.g. economics, medicine, sociology)		
**30. What exposures are studied in parent study?** *List which are randomized and which are non-randomized*		
**31. What are the outcomes in the parent study?** (e.g. myocardial infarction, hypertension, remission)		
**32. In which journal was the parent study published?**		
**33. Funding Source** *Copy & paste funding section of manuscript here*		
**34. Key conclusions of study authors (of pooled study data not single studies).** *From abstract section*		
**35. Notes**		

4. Methods and reporting standards

	Copy and paste related descriptions as stated in report/paper	Location in text (pg & paragraph/fig/table)
**36. Did they discuss issues such as difference in variable definitions, data quality, missing data?**		
**37. Did they take approach(es) to account for differences in variable definitions and data quality across individual cohorts?** *(e.g. any stated information about harmonization efforts (redefining variables) or statistical methods (adopting measurement error methods) studies/cohorts, etc), or did authors deal with missing data within and across studies? (e.g. multilevel multiple imputation, or separate imputation for each dataset, or complete case analysis)*		
**38. How did authors deal with missing data within and across studies?** *(e.g. multilevel multiple imputation, or separate imputation for each dataset, or complete case analysis)*		
**39. Do the authors report testing any of the assumptions required for the analysis methods they have chosen to pool the data? Which ones?** *Copy and paste relevant text describing the tests or reporting the results of those tests, if any, here. If not reported, write "not reported". If unclear, write "unclear".*		
**40a. Approach(es) to account for clustering/heterogeneity at the cohort or study level (whichever units are pooled across)** *(e.g. no adjustment,*		

fixed effects (indicator variable for each cluster), random effects (random effect for each cluster), robust standard errors, etc). Did the authors adopt a one-stage or two-stage approach? (See e.g. Debray et al. PLOS ONE for a description of this)		
**40b. Did the authors explicitly state assumptions for methods to account for clustering/heterogeneity?** *(choice of fixed effects, random effects, ignoring of clustering: simply analyzing all data as if they come from one study)*		
**41a. Which causal methods were used with the pooled data to make causal inferences?** *(e.g. interrupted time series with a control group; comparative study without concurrent controls; instrumental variables; Mendelian randomization; regression discontinuity; interrupted time series, including difference-in-differences estimation; G-estimation; multiple regression adjusting for confounders; propensity score matching; inverse probability of treatment weighting; etc - this is not an exhaustive list)*		
**41b. Justification for method(s) used** *(e.g. "we selected a synthetic control approach because this method is well-suited to situations involving 1 intervention unit, and many controls and may better approximate counterfactual post-intervention outcomes than using any single control or an evenly weighted combination of controls" or "This approach is advantageous, because characteristics of each region, other than the occurrence of the treatment, are unlikely to change*		

appreciably over so short a time period. Thus, each region serves as its own control, allowing us to control for other community-level characteristics that may also be associated with injuries.")		
**41c. Did the authors explicitly state the assumptions required for causal inference methods? Which ones?** (e.g. ignorability, positivity, stable unit treatment value, transitivity, *"no unobserved confounding"* …) *Copy and paste relevant text, if any, here. If not reported, write "not reported". If unclear, write "unclear"*		
**41d. Do the authors report testing any of the assumptions required for the analysis methods they have chosen to deliver causal effects? Which ones?** *Copy and paste relevant text describing the tests or reporting the results of those tests, if any, here. If not reported, write "not reported". If unclear, write "unclear".*		
**41e. For untestable assumptions (e.g. unmeasured confounding), is there anything the authors do to evaluate the plausibility of those assumptions (e.g. negative control exposures or outcomes, quantitative bias analysis)? If yes, which ones?** *Copy and paste relevant text describing the tests or reporting the results of those tests, if any, here. If not reported, write "not reported". If unclear, write "unclear".*		

**42. Did the authors discuss heterogeneity of estimated causal effects and the possible impact on the generalizability of research findings?**		

**43. Notes**		
