# Supplementary material for: Current trends in the application of causal inference methods to pooled longitudinal observational infectious disease studies—A protocol for a methodological systematic review
Source: PLoS One. 2021 Apr 29;16(4):e0250778. doi: 10.1371/journal.pone.0250778 (PMC8084147; doi:10.1371/journal.pone.0250778)
Supplement: Attachment — Submitted filename: Response to Reviewers_ PlosOne_ 09April2021.docx [file pone.0250778.s005.docx]

Reviewer #1: The manuscript reports on a protocol for a methodological review on how causal inference methods are used in analyzing pooled longitudinal observational studies in infections diseases. The protocol is complete and clear. The complete search strategy and data extraction forms are attached as supplementary material.

Major comments
1. I cannot make any guess on how many articles will be retrieved, but I thought that limiting the search to three years (2009, 2014, 2019) may yield a limited number of studies included. Are there fallback solutions to extend the search is the number of studies would be relatively small? (I acknowledge that this leaves the definition of a “relatively small” number unanswered).

Thank you for your comment. There is currently no fallback solution. We actually had too many studies (nearly 8,000 full-text) in a similar review we conducted with a similar protocol but different focus, so we narrowed the project to infectious disease, hoping for a more manageable (smaller) number of full-text articles to review. Thank you for the concern.

2. I also wondered whether the search strategy would be specific enough to spare a lot of screening time. I am not a specialist of building search equations, but I can only underline the risks of screening hundreds (thousands?) of articles on tile and abstract to include a small number at the end.

Yes, we agree that this is a concern. In order to deal with this, we’ve tested the search with several different librarians at UCSF, Harvard and Heidelberg. It looks like there will be around 8,000 title/abstracts. We agree that doing these methodological systematic reviews can be a risk. But we are dedicated to completing and publishing the results of this review. We do hope to learn from this endeavor and also hope that whatever information is retrieved will better inform fellow researchers in our field.

3. I understand specificities of pooled data analyses, in particular regarding the need to account for heterogeneity between cohorts, but to some extent this is not markedly different from heterogeneity between facilities in a multicenter single cohort study (that are excluded), at least from a statistical point-of-view. There may be important differences with e.g. systematically missing data that are not recorded in a specific study that require specific methods (e.g. Resche-Rigon, White. Stat Methods Med Res 2018;27:1634-1649), and the paper also mentions approaches to account .for differences in data quality or definitions. But some more discussion on why focus only on pooled analyses could be given.

This is a good point. We have tried to incorporate additional emphasis on what makes pooled analyses distinct and advantageous compared to multicenter studies, while also acknowledging the similarities. The relevant sections (lines 90-96 and 136-140) now read:

“In population science, a large sample size is required. A large sample size is also generally required to examine rare exposures/treatments, as is often the case with infectious disease research. However, conducting single cohort studies large enough to reach such a large sample size can be too expensive and time-consuming. To overcome this problem, scientists often pool data from numerous studies. While the pooling of both aggregate data (AD) and individual patient data (IPD) are valuable, the pooling of IPD can yield more reliable results than AD(3,4). Additionally, pooled analyses across diverse cohorts can offer greater variability in exposure and outcome measures, thereby enhancing power and the ability to detect meaningful associations.”

“Although similar to multi-center single cohort studies, implementing causal methodologies with IPD from several cohorts involves a different process and can be more complex, particularly when accounting for, e.g., differences in types of variables that are captured and the ways they are measured, more extreme heterogeneity in cohort composition, or missing data (11,12).”

Minor comments
1. The meaning of “pooling” (that I understood as pooling different observational studies together) could be clarified in the abstract.

Thank you for your feedback. We have clarified this succinctly as:

“Pooling (or combining) and analyzing observational, longitudinal data at the individual level facilitates inference through…” in line 37. If the reviewer has another preferred term, we would be happy to incorporate it.

2. Is there really a need for both IV and IVA acronyms? The first one is largely sufficient.

We agree that this distinction was unnecessary. We have deleted the second acronym in line 110. The section now reads, “These causal methods include but are not limited to instrumental variables (IV),…”

Reviewer #2: This manuscript is a protocol for a methodological systematic review the application of causal inference methods to Pooled longitudinal observational infectious disease studies. No statistical meta-analysis will be needed. This study will be a descriptive summary of the publications that applied causal inference methods. It will be interested in looking at the summary rather than this protocol.

Lines 153-154, why do you select studies published at three timepoints instead of all related publications over the last 15 years?

Thank you for your questions. First, we corrected the span to 10 years throughout the manuscript. We restricted the years of study for feasibility given the large number of studies likely to be returned from the search. To address your concern, we have also now included detail in line 180-182 which now reads:

“We will define ‘recent trends’ as the last 10 years (2009-2019), but will, due to capacity, limit the search to include studies published at three timepoints 2009, 2014, and 2019.”

Reviewer #3: Manuscript ID: PONE-D-20-34304
Title: Current Trends in the Application of Causal Inference Methods to Pooled Longitudinal Observational Infectious Disease Studies -- A Protocol for a Methodological Systematic Review

I thank both the Editor and the Authors for the opportunity to review the above-mentioned manuscript.
In brief, this paper reports the protocol for an ambitious review on causal inference methods applied to pooled observational data in infectious disease studies. The paper promises a useful and most-needed systematic review, which will help researchers and clinicians to better understand the respective usages and pros/cons of the different methods under consideration. While I look forward to the results of this systematic review, I have a couple of very minor comments for this protocol. I hope this will help the Authors clarify some points.

***Abstract:
Line 39: The term “individual-level effects” can be confusing for causal inference researchers, as it may refer to “individual treatment effects” (i.e. the causal effect of an exposure/intervention in a particular individual; note, this quantity is unmeasurable.) I suppose the Authors refer to the individual-study-level effects instead;

Thank you very much for noting this point. We have revised the term to clarify that our meaning referred to the level of the exposure variables. The sentence now from lines 37-40 now reads;

“Pooling (or combining) and analyzing observational, longitudinal data at the individual level facilitates inference through increased sample sizes, allowing for joint estimation of study- and individual-level exposure variables, and better enabling the assessment of rare exposures and diseases.”

Line 42: Causal methods can address both/either unmeasured and/or measured confounders;

Thank you. We changed this to include your suggestion. Line 42 now reads, “The adoption of so-called “causal” methods to account for both/either measured and/or unmeasured confounders is an important addition to the methodological toolkit…”

Line 44: I understand a big C-something has been invading our lives since last year, but I find it a bit out of context since this systematic review includes studies from 2009, 2014 and 2019 – and not 2020, as I understand.

Thank you for this comment. We created a SR in early 2020, and our team members felt that 2019 was a fine place to stop because cohorts, most likely, would not be able to harmonize, analyze and publish IPD data so quickly. We also expected that 2020 and 2021 will be anomalous years in scientific publishing that do not reflect the longer-term trends in the use of causal methods that we wished to characterize. We hope that if this review is done rapidly enough, that we can inform those on how to better conduct or report on their methods for COVID-19 studies as well. However, knowing that, we added lines 156-158 to address this. This section now reads,

“In the short-term, we hope that this review can inform those researchers who are currently analysing observational data with the hope of inferring causality. In the long-term we expect that findings from this review will lead to concrete recommendations for the conduct and reporting of causal inference methods applied to pooled, longitudinal, observational data in ID applications.”

***Introduction:
- The Authors could add one or two sentences on the different assumptions needed for the causal methods under consideration (e.g. IV methods addressed unmeasured confounders but require specific IV assumptions [relevance, no shared causes with the outcome and exclusion restriction]; PS methods rely on the assumption of no unmeasured confounders and require positivity);

Thank you for the suggestion. Details have been added to lines 128-137 to address this comment. This section now reads,

“Each of these causal methods has assumptions (or, conditions) which must be satisfied in order for the method to yield reliable results: e.g. for IV, relevance, exclusion restriction, exchangeability, and monotonicity or homogeneity; and for propensity score methods (PS), exchangeability, consistency, and positivity. Some of the assumptions, or conditions, required for these methods are testable. Some of them, though, are untestable, and requires one to evaluate the feasibility of them, often relying on prior literature, theory, causal models, or background knowledge. Discussing the testing of testable assumptions and evaluation of untestable assumptions in the published research article allows the reader to better understand the rigor with which a researcher approached the issue(s).”

- Line 104: The brief one-sentence summary on PS methods is vague and incorrect: PS methods do not necessarily rely on matching (e.g. weighting, standardization, etc. can be applied too) to provide covariate balance;

Thank you for this correction. Details have been added lines 111-113. This section now reads,

“(8); propensity scores (PS), which can be implemented in several ways, including weighting, matching, or subclassification, e.g., to adjust for covariates, allowing the exposed and unexposed to be more comparable…”

- Line 119-120: the sentence “with non-randomized exposure data from RCTs” sounds paradoxical. Could the Authors reformulate?

Thank you for raising that this point was unclear. Here we are referring to study the effects of baseline variables in the RCT that were not subject to randomization. For example, a study may have randomized a drug treatment on a health outcome, but the study participants could also be treated as a cohort to examine the effect of some other baseline exposure variable (e.g. blood pressure) on the outcome. We have rephrased the wording in lines 145-148. It now reads,

“One study has reviewed the application of causal inference methods applied to time-dependent confounders and also examined which questions are being investigated using non-randomized exposure variables in cohort data deriving from RCTs.”

***Methods:
- Line 166: The Authors state that they will include studies that “4) estimated a causal effect related to a stated causal question”. While it sounds fair, I fear that causal questions are unfortunately too rarely explicitly stated in practice (see Hernan MA. The C-Word: Scientific Euphemisms Do Not Improve Causal Inference From Observational Data. Am J Public Health. 2018;108(5):616-619.)

You are absolutely correct. While we have discussed this, and we did not make this explicit in our protocol. Thank you. We have added a new passage to the introduction and we now reference it in the methods (line 194-196).

Lines 98-103 now read,

“In analysing observational data, the famous phrase *‘correlation does not imply causation’* has hindered the use of causal language: academic journals and peers alike often discourage the use of causal terminology. And, although there has been much work done in the field of causal inference with observational data over the last decades(5–7), many authors still use terms that skirt the issue, employing terms like ‘link’ or ‘associated with’(7). However, as some point out, “the proscription against the C-word is harmful to science…”(7).”

And lines 198-200 now read,

“estimated a causal effect related to a stated causal question (or, what is interpreted by reviewers as a research study motivated by causal question; see reference to avoidance of causal language in the introduction),…”

- Amongst the bullet points for the extraction, I would also be interested in the covariate selection for adjustment (e.g. Did the study explicitly specify that covariates adjusted for were confounders [and not mediators] of the causal relationship?)

Thank you for your comment here. This is a very interesting point, and we will take it into consideration. However, we would not like to commit to doing this work by adding it in the protocol. We appreciate the feedback, though, and agree that this would be a very interesting point to examine.

***Other comments:
- I found some typos throughout, and some sentences quite long and heavy to read (in particular, in the introduction).

We have closely reviewed and copyedited the manuscript throughout.

- Some key references about causal inference methods would be appreciated – e.g. Hernan and Robins book or others.

Thank you. We have added a few key citations:

5.	Rubin D. B. Estimating causal effects of treatment in randomized and nonrandomized studies. J Educ Psychol [Internet]. 1974;66(5):688–701. Available from: http://www.fsb.muohio.edu/lij14/420_paper_Rubin74.pdf

6.	Halloran ME, Struchiner CJ, Epidemiology S, Mar N, Halbran ME, Struchiner CJ. Causal Inference in Infectious Diseases. 1995;6(2):142–51.

7.	Hernan M, Robins J. Causal Inference: What If. Boca Raton: Chapman & Hall/CRC; 2020.

**Second Round of Requested Revisions—9 April 2021**

Reviewer #3: I thank the Authors for addressing my previous comments/suggestions. I enjoyed reading the revised manuscript, which I think reads very well.
 I have a very minor remark, which may however have its own importance: something wrong may have happened with the editing of the references in the revised manuscript - some citations have been deleted in the main text (in particular, Introduction). Could the Authors amend this, and double-check/proof-read before submitting?

I have no further comments other than this one. I thank the Authors for their work.

Thank for your constructive feedback and your compliments. We have added the 2 references that were deleted in the last submitted manuscript. We have also proof-read the manuscript again and edits the places where we say that there were errors in the punctuation or writing. Thank you again.
